# Age-adjusted interpretation of biomarkers of renal function and homeostasis, inflammation, and circulation in Emergency Department patients

**DOI:** 10.1038/s41598-022-05485-4

**Published:** 2022-01-28

**Authors:** Bart G. J. Candel, Jamèl Khoudja, Menno I. Gaakeer, Ewoud ter Avest, Özcan Sir, Heleen Lameijer, Roger A. P. A. Hessels, Resi Reijnen, Erik van Zwet, Evert de Jonge, Bas de Groot

**Affiliations:** 1 Department of Emergency Medicine, Leiden University Medical Center, Albinusdreef 2, 2300 RC Leiden, The Netherlands; 2 Department of Emergency Medicine, Máxima Medical Center, De Run 4600, 5504 DB Veldhoven, The Netherlands; 3 Department of Emergency Medicine, Admiraal de Ruyter Hospital, ‘s-Gravenpolderseweg 114, 4462 RA Goes, The Netherlands; 4 Department of Emergency Medicine, University Medical Center Groningen, Hanzeplein1, 9713 GZ Groningen, The Netherlands; 5 Department of Emergency Medicine, Radboud University Medical Center, Houtlaan 4, 6525 XZ Nijmegen, The Netherlands; 6 Department of Emergency Medicine, Medical Center Leeuwarden, Henri Dunantweg 2, 8934 AD Leeuwarden, The Netherlands; 7 Department of Emergency Medicine, Elisabeth-TweeSteden Hospital, Doctor Deelenlaan 5, 5042 AD Tilburg, The Netherlands; 8 Department of Emergency Medicine, Haaglanden Medical Center, Lijnbaan 32, 2512 VA The Hague, The Netherlands; 9 Department of Biostatistics, Leiden University Medical Center, Albinusdreef 2, 2300 RC Leiden, The Netherlands; 10 Department of Intensive Care Medicine, Leiden University Medical Center, Albinusdreef 2, 2300 RC Leiden, The Netherlands

**Keywords:** Geriatrics, Biomarkers

## Abstract

Appropriate interpretation of blood tests is important for risk stratification and guidelines used in the Emergency Department (ED) (such as SIRS or CURB-65). The impact of abnormal blood test values on mortality may change with increasing age due to (patho)-physiologic changes. The aim of this study was therefore to assess the effect of age on the case-mix adjusted association between biomarkers of renal function and homeostasis, inflammation and circulation and in-hospital mortality. This observational multi-center cohort study has used the Netherlands Emergency department Evaluation Database (NEED), including all consecutive ED patients ≥ 18 years of three hospitals. A generalized additive logistic regression model was used to visualize the association between in-hospital mortality, age and five blood tests (creatinine, sodium, leukocytes, C-reactive Protein, and hemoglobin). Multivariable logistic regression analyses were used to assess the association between the number of abnormal blood test values and mortality per age category (18–50; 51–65; 66–80; > 80 years). Of the 94,974 included patients, 2550 (2.7%) patients died in-hospital. Mortality increased gradually for C-reactive Protein (CRP), and had a U-shaped association for creatinine, sodium, leukocytes, and hemoglobin. Age significantly affected the associations of all studied blood tests except in leukocytes. In addition, with increasing age categories, case-mix adjusted mortality increased with the number of abnormal blood tests. In summary, the association between blood tests and (adjusted) mortality depends on age. Mortality increases gradually or in a U-shaped manner with increasing blood test values. Age-adjusted numerical scores may improve risk stratification. Our results have implications for interpretation of blood tests and their use in risk stratification tools and acute care guidelines.

**Trial registration number** Netherlands Trial Register (NTR) NL8422, 03/2020.

## Introduction

In Emergency Department (ED) patients, correct risk stratification is important to early recognize clinical deterioration and to appropriately assign patients to a definitive level of care^1^. Many risk stratification tools, such as SIRS, CURB-65, PIRO, GRACE, Ranson criteria, assign points for abnormal blood test values^2–8^. A previous study showed, however, that risk stratification tools are ineffective at predicting mortality in older ED patients^9^. This may be caused by a different impact of abnormal blood test values on mortality with increasing age due to (patho)-physiological changes^10,11^. For example, older patients normally have higher creatinine levels than younger patients^12^. Thus, in younger patients an increase in plasma creatinine reflects a larger decrease in renal function compared to older patients and may therefore also carry a larger risk.

In addition, due to less physiological reserve, or frailty, with increasing age, an increasing number of abnormal blood test values may carry a larger risk in older patients.

As a result, using age-adjusted risk scores for blood tests may have the potential to improve risk stratification and consequently facilitate early recognition of clinical deterioration. This early recognition may potentially improve outcome of patients with medical conditions who need treatments that are time sensitive.

The aim of the present study is twofold: First, to assess the effect of age on the associations between biomarkers of renal function and homeostasis, inflammation and circulation and in-hospital mortality. Secondly, we investigate the risk of an increasing number of abnormal blood tests per age category.

## Methods

### Study design and setting

This observational multi-center study was conducted in three EDs in the Netherlands, with each approximately 20,000–30,000 ED visits per year. Consecutive ED visits were registered of one tertiary care center (1 January 2017–8 June 2019), and two urban hospitals (1 January 2019–12 January 2020 and 1 January 2017–31 December 2019). This study was registered in the Netherlands Trial Register (ID NL8422) and approved by the medical ethics committee of the Leiden University Medical Center, the Netherlands. All methods were performed in accordance to the principles outlined in the Declaration of Helsinki.

### Selection of participants

All consecutive ED visits of ≥ 18 years were included in this study, if at least one of the blood tests described below had been registered.

### Data collection

Data were collected from the Netherlands Emergency department Evaluation Database (NEED), the national quality registry for EDs in the Netherlands (www.stichting.need.nl). Correspondent to other quality registrations, and in accordance with the “General Data Protection Act”, an informed opt-out procedure was used in the participating hospitals for inclusion in the NEED. Detailed information about the NEED and collected data are available in an earlier publication^13^. We selected biomarkers which represent organ (dys-)function and inflammation because these biomarkers are known to have prognostic or diagnostic value in the ED, are commonly used in risk scores developed for the ED and are routinely assessed in the ED. For example, creatinine reflects renal function, which is the first organ to fail in early disease stages such as sepsis^2–8^. The following routinely measured biomarkers were investigated: creatinine, urea, and sodium (reflecting renal function and homeostasis), C-reactive protein (CRP) and leukocytes (reflecting inflammation), lactate and hemoglobin (reflecting circulation). In Supplemental digital content 1, details of the used laboratory assays and equipment are described.

### Outcome measures

In-hospital mortality (including death in the ED) was the primary outcome measure.

### Sample size estimation

For objective 1, we aimed to adjust for six potential confounders in the multivariable logistic regression analysis. For objective 2, we aimed to adjust for 39 potential confounders as described in the main statistical analyses section. Approximately five to ten events per variable are needed to prevent overfitting in association studies^14^. The NEED contained 148,828 ED visits of patients ≥ 18 years of age. We estimated that in ~ 60% of the ED visits blood tests were performed resulting in ~ 90,000 ED visits which could be used for the analyses. Estimated in-hospital mortality would be ~ 3% of the overall population. Included patients were stratified by age, yielding ~ 90,000/4 age categories = 22,500 patients per age category. On average we would have 0.03 × 22,500 = 675 events per group, sufficient to adjust for the 39 potential confounders.

### Descriptive analysis

Patient characteristics were summarized per age-category (18–50, 51–65, 66–80, and > 80 years)^12^, as mean (SD) for normally distributed data, and median (IQR) for skewed data. Per age category, the percentage of blood test values outside of the reference range were reported.

### Main statistical analyses

For objective 1, our goal was to study how creatinine, sodium, leukocytes, CRP, and hemoglobin are associated with mortality in emergency care, and how these associations changes with age. We used the R package mgcv^15^, to fit a generalized additive logistic regression model (GAM) to the binary outcome in-hospital mortality. In this model, the log odds of the outcome depends in an arbitrary way on all six predictors (five blood parameters and age) and all their interactions. Clearly, such a complex model is overparameterized which could lead to poor, unstable performance if left unaddressed. However, it can be handled as part of the fitting procedure by using a quadratically penalized likelihood type approach. Effectively, this method enforces a smooth dependence of the log odds mortality on the six predictors. To visualize the association between mortality and the six predictors, we generated five graphs which present different views on the same model. In each of the graphs, we vary one of the predictors together with age, while the other four predictors are left constant at “typical” values. These were chosen as follows: creatinine = 80 µmol/L, leukocytes = 8.0 × 10^9^/L, CRP = 10 mg/L, Sodium = 140 mmol/L, and hemoglobin = 9 mmol/L.

This analysis was intended to be descriptive and exploratory, and therefore we did not attempt formal statistical inference in terms of p-values and confidence intervals.

Due to the complexity of the model, we could not add other biomarkers or potential confounders. To assess whether adjustment for other potential confounders affected our results, a second analysis was performed, in which we also studied the biomarkers urea and lactate. Multivariable logistic regression analyses were used with separate models for each biomarker for each age category. We simplified the model by categorizing blood tests, based on expected distribution and reference intervals (see Supplemental digital content 1). The following potential confounders were entered in the models through backward stepwise elimination: age, gender, triage level (non-urgent, urgent, very urgent, most urgent), top ten presenting complaints (Supplemental digital content 2), systolic blood pressure, heart rate, peripheral oxygen saturation, hospital, high dependency care unit admission (yes or no), number of consultations in the ED (0, 1, 2 or > 2) and performed radiological tests (0 or ≥ 1). The models for creatinine, urea, CRP, and leukocytes were adjusted for hemoglobin and sodium. The model for sodium was adjusted for hemoglobin and urea, and the model for hemoglobin was adjusted for sodium and urea. If variation inflation factors (VIF) were below three, multicollinearity was assumed not to be a problem. Triage level, high dependency care unit admission and vital signs were used to adjust for disease severity^9^. Vital signs were categorized in five or six categories to overcome non-linear associations, including a category ‘not measured’ to prevent missing data. In the NEED comorbidities were not registered. The number of consultations in the ED and the number of radiological tests were used as measure of comorbidities/complexity as described previously^16^. If variables were missing, the patient was excluded from the analyses. However, the used potential confounders had almost no missing data in the NEED. After the analyses for each biomarker was performed in all age categories, the analyses were repeated for the pooled data. An interaction term of age*blood test was added to study whether age affected the association between the studied biomarker and mortality. We considered age as an effect modifier if the p-value of the interaction term was < 0.05. Adjusted odds ratio’s (AORs) with 95% Confidence Intervals (95% CI) and predicted probabilities (mean case-mix adjusted mortality) were reported to compare relative risk increases with absolute mortality with changing blood test values.

For objective 2, the number of abnormal blood test values, outside of their reference intervals, was calculated for each age category. The used reference intervals are mentioned in Supplemental digital content 1. We studied the association between the number of abnormal blood tests and mortality with multivariable logistic regression using similar potential confounders as described above.

Data were analyzed using SPSS (Version 25.0) and R version 4.0 (packages foreign, dplyr, mgcv).

## Results

### Patient inclusion and characteristics

Out of the 147,728 ED visits of patients ≥ 18 years, 94,974 patients were included in whom blood tests were performed (flow diagram in Supplemental digital content 3). Mean age of included patients was 60.7 (19.0) years. In-hospital mortality per age category was: 153 (0.6%) patients in age category 18–50 years, 421 (1.8%) patients 51–65 years, 1086 (3.6%) 66–80 years, and 890 (6.3%) patients > 80 years. Biomarkers were more often abnormal in older patients. For example, sodium was in 9.2% abnormal in age-category 18–50 years, and in 25.5% in age-category > 80 years (difference 16.3%; 15.5–17.1%). See Table 1 for all patient characteristics and Supplemental digital content 4 for patient disposition and outcomes.

**Table 1:** Patient characteristics in the total cohort and per age category.

	Total cohort N = 94,974 (100.0%)	18–50 years N = 26,697 (28.1%)	51–65 years N = 23,840 (25.1%)	66–80 years N = 30,257 (31.9%)	> 80 years N = 14,180 (14.9%)
**Demographics N, (%)**					
Age, mean (SD)	60.7 (19.0)	35.5 (9.7)	58.5 (4.3)	72.9 (4.2)	85.7 (3.9)
Sex, female	47,082 (49.6)	14,979 (56.1)	11,013 (46.2)	13,297 (43.9)	7793 (55.0)
**Hospital setting N, (%)**					
Tertiary care centre	28,665 (30.2)	9396 (35.2)	7762 (32.6)	8448 (27.9)	3059 (21.6)
**Top-10 presenting complaints N, (%)**					
Collapse	4001 (4.2)	800 (3.0)	930 (3.9)	1510 (5.0)	761 (5.4)
Extremity problems	5803 (6.1)	1151 (4.3)	1278 (5.4)	1876 (6.2)	1498 (10.6)
Headache	2000 (2.1)	817 (3.1)	511 (2.1)	475 (1.6)	197 (1.4)
Palpitations	3150 (3.3)	554 (2.1)	960 (4.0)	1301 (4.3)	335 (2.4)
Chest pain	11,372 (12.0)	2864 (10.7)	3611 (15.1)	3645 (12.0)	1252 (8.8)
Wounds	1390 (1.5)	430 (1.6)	365 (1.5)	432 (1.4)	163 (1.1)
Feeling unwell	21,268 (22.4)	4095 (15.3)	5417 (22.7)	7801 (25.8)	3955 (27.9)
Abdominal pain	14,745 (15.5)	6875 (25.8)	3643 (15.3)	3197 (10.6)	1030 (7.3)
Dyspnea	12,126 (12.8)	2055 (7.7)	2882 (12.1)	4831 (16.0)	2358 (16.6)
Trauma	2449 (2.6)	859 (3.2)	507 (2.1)	639 (2.1)	444 (3.1)
Miscellaneous	16,670 (17.6)	6197 (23.2)	3736 (15.7)	4550 (15.0)	2187 (15.4)
**Proxies for disease Severity N, (%)**					
Triage level					
Blue/green	18,157 (19.1)	5626 (21.4)	4286 (18.3)	5438 (18.2)	2807 (20.1)
Yellow	41,499 (43.7)	12,236 (46.6)	10.354 (44.1)	12,909 (43.3)	6000 (42.9)
Orange	28,436 (29.9)	7291 (27.7)	7367 (31.4)	9504 (31.9)	4274 (30.6)
Red	5449 (5.7)	1128 (4.3)	1472 (6.3)	1971 (6.6)	878 (6.3)
**Biomarkers of renal function and homeostasis, Median (IQR), [N]**					
Creatinine (μmol/L)	77 (63–97)	69 (59–82)	75 (62–91)	83 (67–106)	91 (72–122)
Urea (mmol/L)	5.8 (4.3–7.9)	4.3 (3.4–5.4)	5.5 (4.4–7.0)	6.7 (5.2–9.0)	8.1 (6.2–11.3)
Sodium (mmol/L)	140 (137–142)	140 (138–142)	140 (137–142)	139 (136–141)	139 (136–141)
**Biomarkers of inflammation, Median (IQR), [N]**					
CRP (mg/L)	10.4 (4.2–47)	7.3 (3.8–32)	10.3 (4.0–49)	12.0 (5.2–50)	12.0 (5.2–50)
Leucocytes (× 10^9^/L)	9.1 (7.0–12.0)	9.3 (7.2–12.1)	8.9 {6.9–11.9)	9.0 (6.9–12.)	9.2 (7.1–12.2)
**Biomarkers of Circulation Median (IQR), [N]**					
Hemoglobin (mmol/L)	8.4(7.6–9.2)	8.7 (7.9–9.3)	8.6 (7.8–9.3)	8.3 (7.4–9.1)	7.9 (7.1–8.7)
Lactate (mmol/L)	1.6 (1.1–2.3)	1.5 (1.0–2.2)	1.5 (1.1–2.3)	1.6 (1.1–2.4)	1.6 (1.1–2.4)
**Proxies for comorbidity and complexity N, (%)**					
Radiological test^a^	58,780 (61.9)	13,713 (51.4)	14,503 (60.8)	19,922 (65.8)	10,642 (75.0)
**Number of consultations**					
None	32.791 (34.5)	10,600 (39.8)	8355 (35.0)	9649 (31.9)	4187 (29.5)
1	54.814 (57.7)	14,233 (53.4)	13,723 (57.6)	18,215 (60.2)	8643 (61.0)
2	6293 (6.6)	1596 (6.0)	1491 (6.3)	2046 (6.8)	1160 (8.2)
> 2	929 (1.0)	223 (0.8)	221 (0.9)	306 (1.0)	179 (1.3)

Blood tests used in risk stratification and acute care guidelines were selected, reflecting biomarkers of renal function and homeostasis, inflammation, and circulation.

Number of patients in the total cohort was for creatinine (N = 89,784), urea (N = 88,816), sodium (N = 91,617) and lactate (N = 13,717), leukocytes (N = 91,136), CRP (N = 78,085) and haemoglobin (N = 92,304).

*N* number, *SD* standard deviation, *GP* General Practitioner, *IQR* interquartile range, *CRP* C-Reactive Protein, *μmol/L* micromole per litre, *mmol/L* millimole per litre, *mg/L* milligram per litre.

^a^If one or more of the following radiological tests were performed: ultrasound, radiography, and computer-tomography.

### Main results

**Figure 1:**
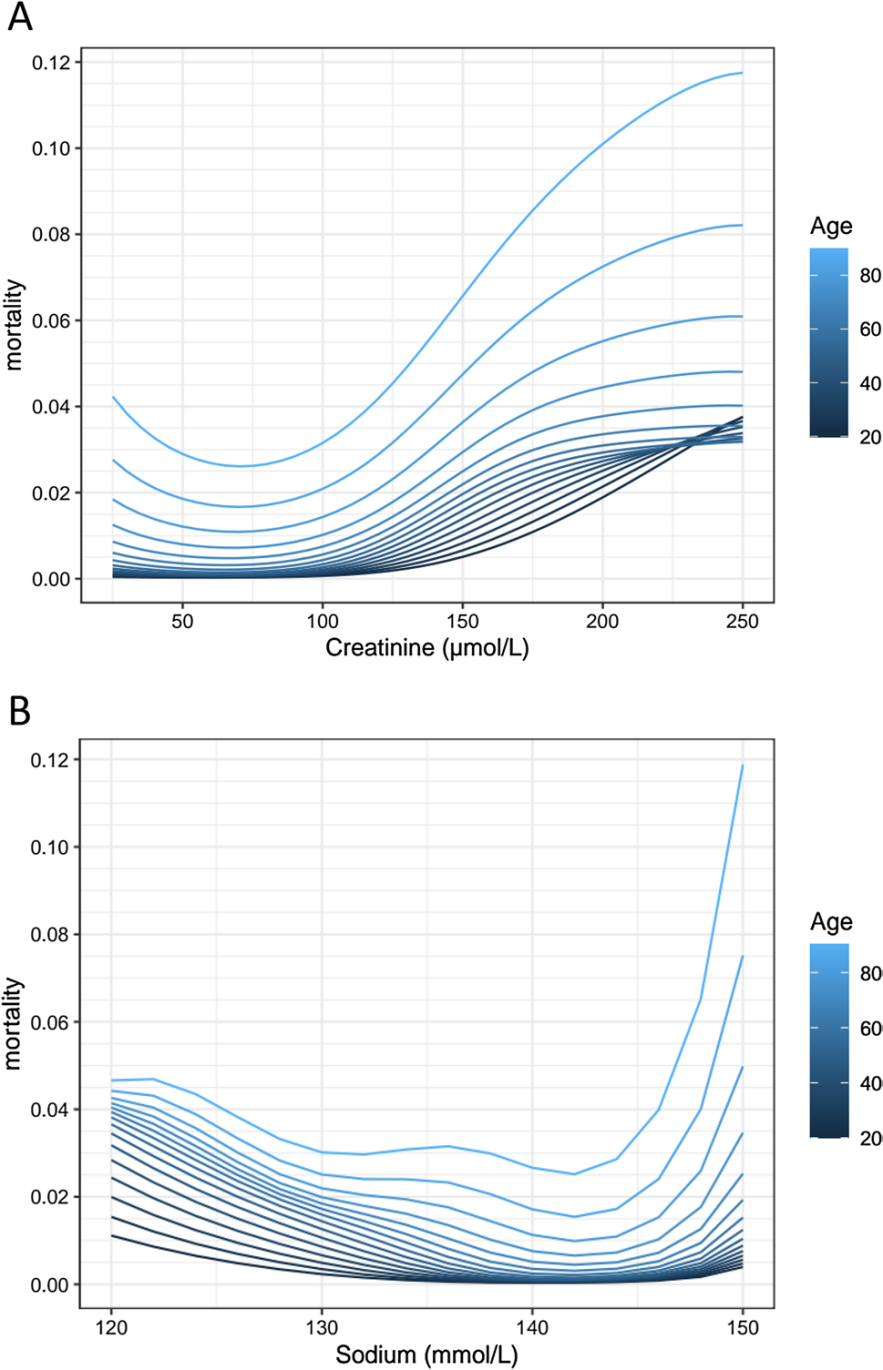
The associations between creatinine (**A**), sodium (**B**) and mortality and age are shown using a generalized additive logistic regression model. This model included five biomarkers and age. The four biomarkers that are not shown in the panel were left constant at ‘normal’ values. These were chosen as follows: creatinine = 80 µmol/L, leukocytes = 80 × 10^9/L, CRP = 10 mg/L, Sodium = 140 mmol/L, and hemoglobin = 9 mmol/L. Mortality is shown as mean predicted mortality risk (between 0 and 1).

**Figure 2:**
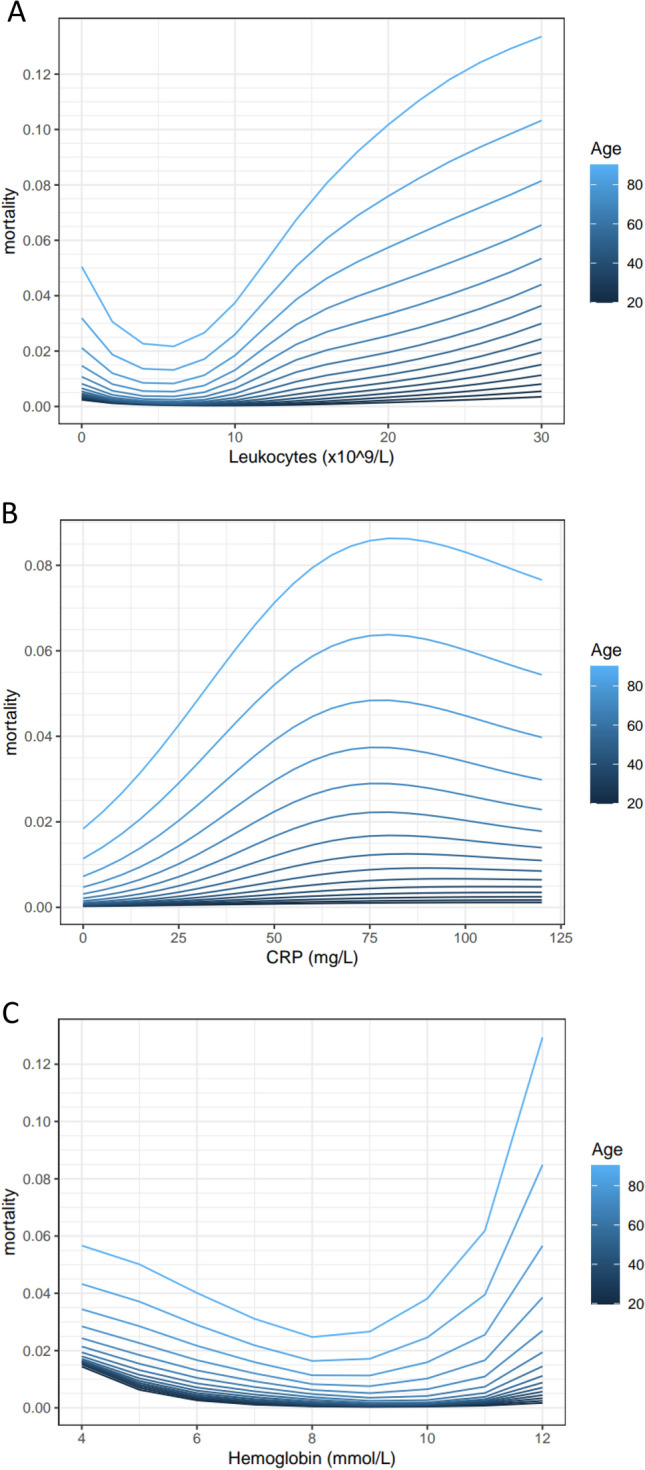
The associations between leukocytes (**A**), C-reactive Protein (**B**), hemoglobin (**C**) and mortality and age are shown using a generalized additive logistic regression model. This model included five biomarkers and age. The four biomarkers that are not shown in the panel were left constant at ‘normal’ values. These were chosen as follows: creatinine = 80 µmol/L, leukocytes = 8.0 × 10^9/L, CRP = 10 mg/L, Sodium = 140 mmol/L, and hemoglobin = 9 mmol/L. Mortality is shown as mean predicted mortality risk (between 0 and 1).

For objective 1, the associations between biomarkers and mortality are represented in Figs. 1 and 2. Creatinine, sodium, leukocytes, and hemoglobin had U-shaped associations with mortality, while mortality increased gradually with increasing CRP. In these figures it is also shown that the absolute risk for mortality increased more in older compared to younger patients with abnormal plasma values.

In the additional analyses, in which we also studied urea and lactate, relative risks (AORs) were presented (see Table 2). Both biomarkers of renal function (creatinine and urea) had a more marked increase in AORs in patients 18–50 years with higher plasma concentrations. AORs started to increase below 136–145 mmol/L in 18–50 years and below 126–130 mmol/L in > 80 years. Mortality increased gradually for CRP, with more marked increase in patients 18–50 years for plasma levels of 51–100 mg/L. Hemoglobin had a U-shaped association with mortality especially in older patients. A low hemoglobin was associated with higher mortality in younger compared to older patients. For lactate, mortality increased gradually in all age categories.

**Table 2:** Adjusted odds ratios with 95% confidence intervals for the association of categorized urea, creatinine, sodium, leukocytes, C-Reactive Protein, hemoglobin and lactate and in-hospital mortality in age-categories.

	Total cohort AOR (95% CI)	18–50 years AOR (95% CI)	51–65 years AOR (95% CI)	66–80 years AOR (95% CI)	> 80 years AOR (95% CI)	p-value
**Biomarkers of renal function and homeostasis**						
Urea (mmol/L)						**0.03**
0–5.0^a^	1.0	1.0	1.0	1.0	1.0	
5.1–10.0	1.7 (1.5–2.0)	2.5 (1.5–4.0)	1.7 (1.3–2.3)	1.5 (1.2–1.9)	1.8 (1.2–2.6)	0.18
10.1–15.0	3.1 (2.6–3.7)	5.7 (2.6–12.2)	3.3 (2.2–5.0)	3.0 (2.3–4.0)	2.9 (1.9–4.3)	< 0.01
> 15.0	5.3 (4.4–6.3)	15.5 (8.3–29.1)	3.4 (2.3–5.0)	5.0 (3.8–6.5)	5.9 (4.0–8.7)	0.10
Creatinine (µmol/L)						**0.01**
0–50	1.5 (1.2–1.8)	1.5 (0.7–2.9)	1.5 (1.0–2.1)	1.4 (1.1–1.9)	1.5 (1.0–2.1)	0.35
51–100^a^	1.0	1.0	1.0	1.0	1.0	
101–150	1.5 (1.3–1.6)	2.9 (1.7–4.8)	1.9 (1.4–2.6)	1.4 (1.2–1.6)	1.3 (1.1–1.6)	**< 0.01**
> 150	2.6 (2.3–2.9)	5.2 (3.1–8.9)	2.3 (1.7–3.2)	2.4 (2.0–2.8)	2.7 (2.2–3.3)	**0.02**
Sodium (mmol/L)						< 0.01
> 145	2.2 (1.8–2.8)	2.0 (0.9–4.4)	2.1 (1.2–3.7)	1.9 (1.3–2.8)	2.6 (1.8–3.8)	0.96
136–145^a^	1.00	1.0	1.0	1.0	1.0	
131–135	1.4 (1.3–1.6)	2.7 (1.6–4.5)	1.9 (1.5–2.6)	1.5 (1.2–1.8)	1.0 (0.8–1.2)	**< 0.01**
125–130	1.7 (1.4–2.1)	2.5 (1.1–6.0)	2.1 (1.4–3.3)	2.0 (1.5–2.6)	1.2 (0.8–1.7)	**0.02**
< 125	2.8 (2.2–3.7)	6.7 (2.6–17.2)	4.4 (2.5–7.6)	2.6 (1.7–4.0)	2.0 (1.2–3.3)	**0.01**
**Biomarkers of inflammation**						
Leucocytes (× 10^9^/L)						0.47
0–4.0	2.4 (2.0–3.0)	3.8 (2.7–8.6)	1.7 (1.1–2.8)	2.3 (1.7–3.1)	2.8 (1.8–4.4)	
4.1–8.0^a^	1.0	1.0	1.0	1.0	1.0	
8.1–12.0	1.3 (1.2–1.5)	1.3 (0.8–2.4)	1.2 (0.9–1.7)	1.3 (1.1–1.6)	1.4 (1.1–1.8)	
12.1–16.0	1.7 (1.5–2.0)	2.2 (1.2–4.1)	1.7 (1.2–2.4)	1.7 (1.4–2.1)	1.8 (1.4–2.2)	
16.1–20.0	2.2 (1.8–2.6)	2.1 (1.0–4.4)	2.6 (1.7–3.8)	2.0 (1.5–2.6)	2.2 (1.6–2.9)	
> 20.0	2.7 (2.3–3.2)	4.9 (2.5–9.8)	2.1 (1.4–3.2)	2.5 (2.0–3.2)	3.0 (2.2–4.1)	
CRP (mg/L)						**< 0.01**
0–50^a^	1.0	1.0	1.0	1.0	1.0	
51–100	1.5 (1.3–1.7)	2.4 (1.3–4.3)	2.0 (1.4–2.8)	1.7 (1.4–2.1)	0.9 (0.7–1.2)	**< 0.01**
101–150	1.9 (1.6–2.3)	1.8 (0.8–4.0)	1.8 (1.2–2.7)	2.2 (1.7–2.8)	1.7 (1.3–2.3)	0.77
> 150	2.4 (2.1–2.7)	2.4 (1.3–4.4)	2.9 (2.2–4.0)	2.4 (2.0–2.9)	2.1 (1.7–2.7)	0.21
**Biomarkers of circulation**						
Hemoglobin (mmol/L)						**< 0.01**
> 9.0^a^	1.0	1.0	1.0	1.0	1.0	
7.1–9.0	1.1 (1.0–1.2)	1.0 (0.6–1.5)	1.4 (1.0–1.9)	1.2 (1.0–1.4)	0.8 (0.7–1.0)	0.18
6.1–7.0	1.5 (1.3–1.7)	2.2 (1.1–4.1)	2.1 (1.4–3.0)	1.4 (1.1–1.8)	1.2 (0.9–1.5)	**< 0.01**
< 6.0	1.7 (1.4–2.0)	2.8 (1.5–5.4)	2.2 (1.5–3.3)	1.8 (1.4–2.4)	1.1 (0.8–1.5)	**< 0.01**
Lactate (mmol/L)						
0–2.0^a^	1.0	1.0	1.0	1.0	1.0	**< 0.01**
2.1–4.0	1.7 (1.4–2.1)	3.4 (1.2–9.6)	2.3 (1.3–4.1)	1.5 (1.1–2.2)	1.7 (1.2–2.5)	0.31
4.1–6.0	3.3 (2.4–4.5)	8.0 (2.0–32.3)	4.2 (1.8–9.7)	2.4 (1.4–3.9)	4.1 (2.4–7.1)	0.44
> 6.0	10.2 (7.6–13.4)	30.7 (11.0–85.6)	14.7 (7.4–28.9)	8.5 (5.4–13.5)	8.7 (4.7–15.9)	**< 0.01**

The following potential confounders were entered in the model through backward stepwise regression: age, gender, triage category (green/blue, yellow, orange, red), top ten presenting complaints, hospital, systolic blood pressure, heart rate, peripheral oxygen saturation, high dependency care unit admission, number of consultations in the ED (0, 1, 2 or > 2), performed radiological tests and blood tests.

Number of patients in the total cohort was for creatinine (N = 89,784), urea (N = 88,816), sodium (N = 91,617) and lactate (N = 13,717), leukocytes (N = 91,136), CRP (N = 78,085) and hemoglobin (N = 92,304).

*AOR* Adjusted Odds Ratio, *95 CI* 95% confidence interval, *CRP* C-reactive Protein.

The p-value is presented from the interaction term of age*biomarker which was added in the analyses. Values in bold are statistically significant.

^a^Used as reference category in the multivariable logistic regression analyses.

Alternatively, we tested whether age affected the association between biomarkers and mortality using an interaction term. As shown by the significant interaction terms, age significantly affected the associations between urea (p = 0.03), creatinine (p < 0.01), sodium (p < 0.01), hemoglobin (p < 0.01), CRP (p < 0.01) and lactate (p < 0.01) and mortality, but not between leukocytes and mortality (p = 0.47). Table 2 shows in which blood test categories the risk on mortality was affected by age.

Supplemental digital content 5 shows the associations between biomarker categories and mortality in all four age categories, corrected for potential confounders like disease severity and complexity. The results are comparable with the main analyses shown in Figs. 1 and 2, with larger absolute increases of mortality in older patients with abnormal biomarker values.

**Figure 3:**
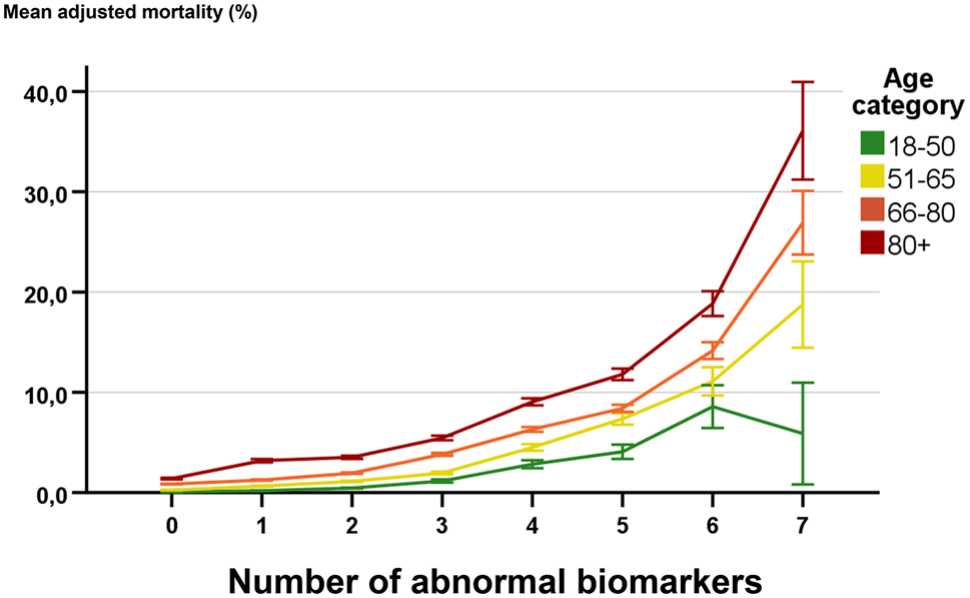
The association between the number of abnormal biomarkers (outside of the commonly used reference ranges) and mortality in different age categories. Mortality is shown as mean predicted mortality risk in percent (between 0 and 100%).

For objective 2, Fig. 3 shows that the risk for absolute case-mix adjusted mortality had larger increases in older patients with an increasing number of abnormal blood test values. Older patients more often had abnormal blood test values (see Supplemental digital content 6).

## Discussion

The present study has two main findings. First, mortality risk for ED patients deteriorates gradually or in a U-shaped fashion with most blood tests. Secondly, the association between blood tests and (adjusted) mortality depends on age, whereas mortality risk is affected most by deviating biomarker levels in younger patients. Age should therefore be considered as an effect modifier rather than a covariate in future prediction models.

In ED-patients, case-mix adjusted mortality increased gradually with increasing urea, CRP, and lactate values, and with the number of abnormal blood tests. For creatinine, sodium, leukocytes, and hemoglobin we found U-shaped associations. These findings correspond to previous studies in which blood tests had similar unadjusted associations with relevant clinical outcomes^17,18^. Several studies used biomarkers in prediction models for the ED^19–27^, with similar associations for mortality. However, to the best of our knowledge, none of these studies investigated the effect of age on the association between biomarkers and outcome. As we showed, the associations between blood tests and case-mix adjusted outcomes changed with age, suggesting that age should be considered as an effect modifier rather than a covariate, possibly because reference intervals of biomarkers change with age. For example, sodium and hemoglobin levels decline with increasing age in a healthy population^28,29^, which may explain our findings that low hemoglobin and low sodium have higher odds on mortality in younger compared to older patients. For urea and creatinine, the AORs for mortality were highest in younger patients, which is in accordance with higher reference values for creatinine and urea in older people^12^. Although younger patients, compared with older patients, had more marked increases in AORs for mortality with deviating urea, creatinine, sodium, CRP, hemoglobin, and lactate, the absolute mortality increase was highest in older patients. This can be explained by the higher overall risk for mortality in older patients, irrespective of the value of plasma biomarker levels.

Abnormal biomarkers are more often present in older patients. In addition, case-mix adjusted mortality had larger absolute increases in older compared to younger patients with an increasing number of abnormal biomarker values, which has not been studied before. The number of abnormal biomarkers had a gradually increasing association with mortality as described before^30^.

Our results imply that risk stratification tools and acute care guidelines for the ED could be improved by incorporating the absolute risk per age category. For example, in current practice, using a biomarker score based on abnormal blood test values, both younger and older patients may be considered as low risk with two abnormal blood tests. However, a mortality risk increase of 100% compared to baseline risk, may result in for example 10% mortality in older patients (baseline risk 5%) and only 2% mortality in younger patients (baseline risk 1%). As a result, older patients may be considered as high risk with similar biomarker values while younger patients are at low or intermediate risk. By using different risk scores for age categories based on absolute measures of mortality, risk stratification may be improved which may lead to better recognition of disease severity, better disposition decisions and consequently lower mortality.

Another finding from our study is that the association between biomarker levels and mortality is gradual with a linear or U-shaped relation. Consequently, risk stratification can be improved by using numerical scores rather than a single cut-off for every biomarker^2,4–8^, as is currently done in the ICU with the Acute Physiology And Chronic Health Evaluation (APACHE) models, in which more points are assigned if blood tests deviate more from the reference range^31^. Although a single cut-off for each biomarker is commonly used in current risk stratification tools, such as the CURB-65 or the SIRS^2,4–8^, our findings suggest not to use a single cut-off for any blood test.

Although this study has its strengths, like the large sample size in multiple EDs, there are also limitations. First, the NEED lacks information about comorbidities. Instead, we had to use proxies known to be associated with comorbidities and complexity to overcome this^16^. Second, inherent to retrospective studies, our study could have been subjected to documentation errors, although this was largely minimized by automatization. Finally, we had no follow-up data, so we had to assume that discharged patients did not experience the composite outcome.

In summary, the prognostic value of blood tests changes with age and mortality risk deteriorates gradually or in a U-shaped fashion with most blood tests for ED patients. Mortality risk is affected mostly by deviating biomarker levels in younger patients. However, due to higher baseline risk in older patients, small changes in biomarker values lead to high mortality. Furthermore, age affected the association between the number of abnormal blood test values and mortality. Our results have implications for interpretation of biomarkers and their use in existing risk stratification tools and guidelines in the ED. Future studies should develop risk stratification tools for ED patients using age-adjusted numerical scores based on absolute risks.

## Supplementary Information

Supplementary Legends.

Supplementary Information 1.

Supplementary Information 2.

Supplementary Information 3.

Supplementary Information 4.

Supplementary Information 5.

Supplementary Information 6.
