# Supplementary material for: Age-adjusted interpretation of biomarkers of renal function and homeostasis, inflammation, and circulation in Emergency Department patients
Source: Sci Rep. 2022 Jan 28;12:1556. doi: 10.1038/s41598-022-05485-4 (PMC8799641; doi:10.1038/s41598-022-05485-4)
Supplement: Supplementary file 1 — Supplementary Legends. [file 41598_2022_5485_MOESM1_ESM.docx]

**LEGENDS TO SUPPLEMENTAL DIGITAL CONTENTS.**

**Supplemental digital content 1.** The reference values provided by the hospital laboratories for all studied blood tests, and the type of kit/device that is used.

**Supplemental digital content 2.** An overview of the merged list of 51 presenting complaints, consisting of the Manchester Triage System (MTS) and the Dutch Triage standard (NTS). The top ten presenting complaints is used for case-mix correction in this study.

**Supplemental digital content 3.**  Patient flow diagram of the study.

**Supplemental digital content 4.** Disposition and outcome of included ED patients per age category in whom biomarkers were assessed.

**Supplemental digital content 5.** Adjusted odds ratios for a composite outcome of in-hospital mortality and Medium Care or Intensive Care Unit admission, as a function of biomarkers of renal function and homeostasis (urea, creatinine, and sodium), inflammation (C-reactive protein and leukocytes), and circulation (hemoglobin and lactate) in different age categories. The following potential confounders were entered in the model through backward stepwise regression: age, gender, proxies for disease severity (triage level (green/blue, yellow, orange, red), vital signs, ICU admission, proxies for comorbidities/complexity (number of consultations in the ED (0, 1, 2 or >2), performed radiological tests), top ten presenting complaints, hospital, and blood tests.

Number of patients in the total cohort was for creatinine (N=89,784), urea (N=88,816), sodium (N=91,617) and lactate (N=13,717), leukocytes (N=91,136), CRP (N=78,085) and hemoglobin (N=92,304).

*Means that age affected the association between the biomarker category and mortality.

**Supplemental digital content 6.** The number of abnormal biomarkers per age category.
