# Supplementary material for: Age-adjusted interpretation of biomarkers of renal function and homeostasis, inflammation, and circulation in Emergency Department patients
Source: Sci Rep. 2022 Jan 28;12:1556. doi: 10.1038/s41598-022-05485-4 (PMC8799641; doi:10.1038/s41598-022-05485-4)
Supplement: Supplementary file 2 — Supplementary Information 1. [file 41598_2022_5485_MOESM2_ESM.docx]

**SUPPLEMENTAL DIGITAL CONTENT 1**

| **Blood test** | **Reference values** | **Device** | **Kit** |
| --- | --- | --- | --- |
| Urea | 2.5-7.5mmol/L (men)  2.5-6.4 mmol/L (women) | Cobas 8000 | Roche |
| Creatinine | 60-110 µmol/L (men)  49-90 µmol/L (women) | Cobas 8000 | Roche |
| Sodium | 135-145 mmol/L | Cobas 8000 | Roche |
| Lactate | 0.5-2.2 mmol/L | ABL90 | Radiometer |
| Haemoglobin | 8.5-11.0mmol/L (men)  7.5-10.0 mmol/L (women) | XN9100 / Cell-Dyn Sapphire | Sysmex, Abbott |
| Leukocytes | 4.0-10.0 x 10^9/L | XN9100 / Cell-Dyn Sapphire | Sysmex, Abbott |
| C-reactive Protein | <5.0 mg/L/  <6.0mg/L | Cobas 8000 | Roche |
