# Supplementary material for: Age-adjusted interpretation of biomarkers of renal function and homeostasis, inflammation, and circulation in Emergency Department patients
Source: Sci Rep. 2022 Jan 28;12:1556. doi: 10.1038/s41598-022-05485-4 (PMC8799641; doi:10.1038/s41598-022-05485-4)
Supplement: Supplementary file 3 — Supplementary Information 2. [file 41598_2022_5485_MOESM3_ESM.docx]

**Supplemental digital content 2**

| The Manchester Triage System (MTS) was used in the tertiary care center, and consisted of 51 possible chief complaints. The Dutch Triage Standard (NTS) consisted of 50 possible triage chief complaints and was used in the two urban hospitals. The MTS and NTS were merged into one chief complaints list. The MTS, NTS and the merged chief complaints list are shown below. If chief complaints did not match, a new chief complaint was made. In total the merged list contained 51 different chief complaints. The top ten chief complaints were used in the present study to adjust the primary associations of interest (feeling unwell; abdominal pain; dyspnea; chest pain; extremity problems; collapse; trauma; palpitations; wounds; headache, and a miscellaneous category containing all the other chief complaints. | | |  |
| --- | --- | --- | --- |
| **Merged chief complaints**  **(N=51)** | **MTS chief complaints**  **(N=51)** | **NTS chief complaints**  **(N=50)** | |
| Abdominal pain | Abdominal pain in adults  Abdominal pain in children | Abdominal pain in adults  Abdominal pain in children | |
| Abscesses & local infections | Abscesses and local infections |  | |
| Allergy, bites & stings | Allergy  Bites and stings | Allergic reaction and stings | |
| Apparently drunk | Apparently drunk |  | |
| Assault | Assault |  | |
| Asthma | Asthma |  | |
| Back pain | Back pain | Back pain | |
| Behaving strangely & suicidal | Behaving strangely | Behaving strangely or suicidal | |
| Breast infection |  | Breast infection | |
| Burns & scalds | Burns and scalds | Burns and scalds | |
| Chest pain | Chest pain | Chest pain | |
| Collapse | Collapsed adult | Collapse  Dizziness | |
| Constipation |  | Constipation | |
| Coughing |  | Coughing | |
| Crying baby | Crying baby |  | |
| Dental problems | Dental problems | Dental problems | |
| Diabetes | Diabetes | Diabetes | |
| Diarrhea & vomiting | Diarrhea and vomiting | Diarrhea  Vomiting | |
| Dyspnea | Shortness of breath in adults  Shortness of breath in children | Shortness of breath | |
| Ear problems | Ear problems | Ear problems | |
| Exposure to chemicals | Exposure to chemicals |  | |
| Extremity problems | Limb problems | Arm problems  General/limb trauma  Leg problems | |
| Eye problems | Eye problems | Eye problems | |
| Facial problems | Facial problems | Facial trauma  Nosebleed | |
| Falls | Falls |  | |
| Feeling unwell | Unwell adult  Unwell child | Fever in adults  Fever in children  Neurological failure  Unwell adult  Unwell child | |
| Fits | Fits | Fits | |
| Foreign body | Foreign body | Foreign body | |
| Gastro-intestinal (GI) bleeding | Gastro-intestinal (GI) bleeding |  | |
| Genital problems | Testicular pain | Genital problems | |
| Headache | Headache | Headache | |
| Implantable Cardioverter Defibrillator (ICD) |  | Implantable Cardioverter Defibrillator (ICD) | |
| Irritable child | Irritable child |  | |
| Limping child | Limping child |  | |
| Major incidents – primary | Major incidents – primary |  | |
| Mental illness | Mental illness |  | |
| Near-drowning |  | Near-drowning | |
| Neck pain | Neck pain | Neck problems  Neck trauma | |
| Overdose & poisoning | Overdose and poisoning | Poisoning | |
| Palpitations | Palpitations | Palpitations | |
| Per vaginum (VP) bleeding | Per vaginum (VP) bleeding | Per vaginum (VP) bleeding | |
| Pregnancy | Pregnancy | Childbirth | |
| Rashes | Rashes | Rashes | |
| Rectal problems |  | Rectal problems | |
| Self-harm | Self-harm |  | |
| Sexually acquired infection | Sexual acquired infection |  | |
| Throat problems | Sore throat | Throat problems | |
| Trauma | Head injury  Torso injury  Major trauma | Head trauma  Thorax trauma  Abdominal trauma  Back trauma | |
| Urinary problems | Urinary problems | Urinary problems | |
| Worried parent | Worried parent |  | |
| Wounds | Wounds | Wounds | |

Abbreviations: MTS=Manchester Triage System, NTS= Dutch Triage Standard, N= number
