# Supplementary material for: Age-adjusted interpretation of biomarkers of renal function and homeostasis, inflammation, and circulation in Emergency Department patients
Source: Sci Rep. 2022 Jan 28;12:1556. doi: 10.1038/s41598-022-05485-4 (PMC8799641; doi:10.1038/s41598-022-05485-4)
Supplement: Supplementary file 4 — Supplementary Information 3. [file 41598_2022_5485_MOESM4_ESM.pptx]

## Slide 1
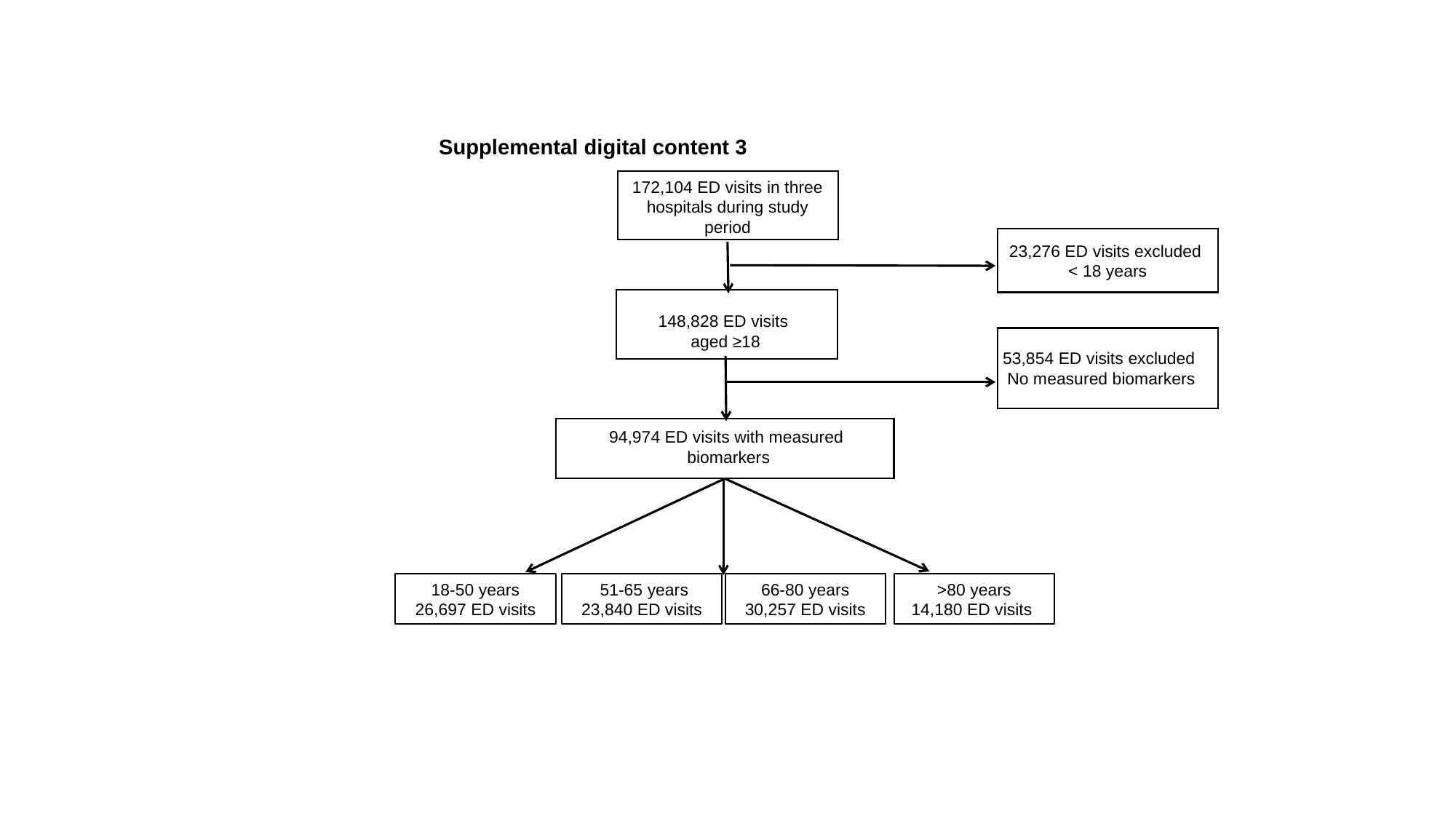

Supplemental digital content 3
172,104 ED visits in three
hospitals during study period
23,276 ED visits excluded
< 18 years
148,828 ED visits
aged ≥18
53,854 ED visits excluded
No measured biomarkers
94,974 ED visits with measured
 biomarkers
>80 years
14,180 ED visits
18-50 years
26,697 ED visits
 51-65 years
23,840 ED visits
66-80 years
30,257 ED visits
