# Supplementary material for: Age-adjusted interpretation of biomarkers of renal function and homeostasis, inflammation, and circulation in Emergency Department patients
Source: Sci Rep. 2022 Jan 28;12:1556. doi: 10.1038/s41598-022-05485-4 (PMC8799641; doi:10.1038/s41598-022-05485-4)
Supplement: Supplementary file 5 — Supplementary Information 4. [file 41598_2022_5485_MOESM5_ESM.docx]

**Supplemental digital content 4**

**Disposition and outcome of included ED patients per age category in whom biomarkers were assessed.**

|  | **Total cohort**  N=94,974 | **18-50 years**  N= 26,697 | **51-65 years**  N=23,840 | **66-80 years**  N=30,257 | **>80 years**  N=14,180 |
| --- | --- | --- | --- | --- | --- |
| **Patient disposition N (%)** | |  |  |  |  |
| Discharged home | 33,711 (35.5) | 13,412 (50.2) | 8901 (37.3) | 8408 (27.8) | 2990 (21.1) |
| Discharged outpatient clinic | 4121 (4.3) | 1554 (5.8) | 1032 (4.4) | 1113 (3.7) | 422 (3.0) |
| Admission to ward | 48,849 (51.4) | 10,093 (38.2) | 11,711 (49.5) | 17,651 (58.3) | 9394 (66.2) |
| Admission to MCU/CCU | 3407 (3.6) | 423 (1.6) | 984 (4.2) | 1403 (4.6) | 597 (4.2) |
| Admission to ICU | 1799 (1.9) | 444 (1.7) | 507 (2.1) | 679 (2.2) | 169 (1.2) |
| Transfer to a different hospital | 2103 (2.2) | 405 (1.5) | 448 (1.9) | 740 (2.4) | 510 (3.6) |
| Died in the ED | 185 (0.2) | 24 (0.1) | 41 (0.2) | 75 (0.2) | 45 (0.3) |
| Missing | 741 (0.01) | 510 (0.01) |  | 179 (0.01) | 52 (<0.01) |
| **Outcome N (%)** |  |  |  |  |  |
| In-hospital mortality | 2550 (2.7) | 153 (0.6) | 421 (1.8) | 1086 (3.6) | 890 (6.3) |
| Abbreviations: N= number, ED= Emergency Department, CCU = Coronary Care Unit, MCU = Medium Care Unit, ICU = Intensive Care Unit, LOS =Length of Stay, IQR = Inter Quartile Range. | | | | | |
