# Supplementary material for: Age-adjusted interpretation of biomarkers of renal function and homeostasis, inflammation, and circulation in Emergency Department patients
Source: Sci Rep. 2022 Jan 28;12:1556. doi: 10.1038/s41598-022-05485-4 (PMC8799641; doi:10.1038/s41598-022-05485-4)
Supplement: Supplementary file 6 — Supplementary Information 5. [file 41598_2022_5485_MOESM6_ESM.pptx]

## Slide 1
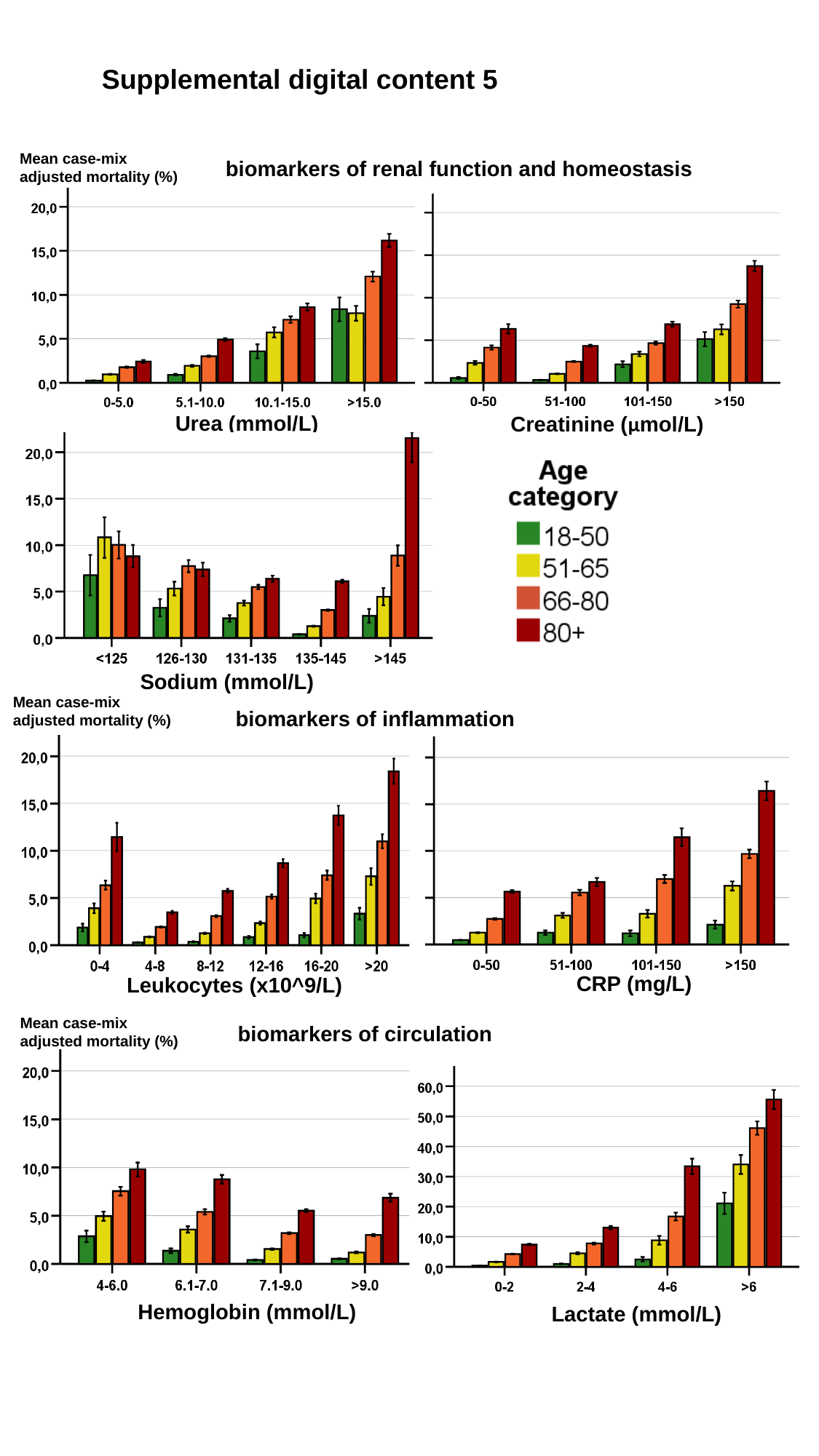

Supplemental digital content 5
Mean case-mix
adjusted mortality (%)
biomarkers of renal function and homeostasis
Urea (mmol/L)
Creatinine (μmol/L)
Sodium (mmol/L)
Mean case-mix
adjusted mortality (%)
biomarkers of inflammation
CRP (mg/L)
Leukocytes (x10^9/L)
Mean case-mix
adjusted mortality (%)
biomarkers of circulation
Hemoglobin (mmol/L)
Lactate (mmol/L)
