# Supplementary material for: Age-adjusted interpretation of biomarkers of renal function and homeostasis, inflammation, and circulation in Emergency Department patients
Source: Sci Rep. 2022 Jan 28;12:1556. doi: 10.1038/s41598-022-05485-4 (PMC8799641; doi:10.1038/s41598-022-05485-4)
Supplement: Supplementary file 7 — Supplementary Information 6. [file 41598_2022_5485_MOESM7_ESM.docx]

|  | 18-50 years | 51-65 years | 66-80 years | >80 years |
| --- | --- | --- | --- | --- |
| Number of abnormal blood test values N (%) | | |  |  |
| 0 | 7478 (28.0) | 5368 (22.5) | 4868 (16.1) | 1410 (9.9) |
| 1 | 8258 (30.9) | 5882 (24.7) | 5799 (19.2) | 2200 (15.5) |
| 2 | 6026 (22.6) | 5200 (21.8) | 6194 (20.5) | 2800 (19.7) |
| 3 | 2949 (11.0) | 3569 (15.0) | 5497 (18.2) | 2974 (21.0) |
| 4 | 1256 (4.7) | 2171 (9.1) | 4273 (14.1) | 2473 (17.4) |
| 5 | 535 (2.0) | 1138 (4.8) | 2516 (8.3) | 1631 (11.5) |
| 6 | 128 (0.7) | 448 (1.9) | 960 (3.2) | 610 (4.3) |
| 7 | 17 (0.1) | 64 (0.3) | 150 (0.5) | 82 (0.6) |
| Abnormal Creatinine, N (%) | 4048 (16.4) | 5692 (25.1) | 9498 (33.0) | 6035 (44.3) |
| Abnormal Urea, N (%) | 3852 (15.7) | 6096 (27.0) | 12,677 (44.5) | 8544 (64.8) |
| Abnormal Sodium, N (%) | 2314 (9.2) | 3731 (16.1) | 6255 (21.2) | 3525 (25.5) |
| Abnormal CRP, N (%) | 8880 (63.6) | 9852 (74.1) | 13,888 (77.5) | 6599 (81.4) |
| Abnormal leukocytes, N (%) | 11,268 (44.4) | 9862 (43.0) | 12,635 (43.4) | 5924 (43.2) |
| Abnormal Hemoglobin, N (%) | 5405 (21.1) | 7051 (30.4) | 12,298 (41.6) | 6771 (48.8) |
| Abnormal Lactate, N (%) | 1522 (33.2) | 1709 (34.1) | 2665 (35.0) | 1370 (36.3) |

**Supplemental digital content 6: Number of abnormal blood test values per age category**

Number of patients in the total cohort was for creatinine (N=89,784), urea (N=88,816), sodium (N=91,617) and lactate (N=13,717), leukocytes (N=91,136), CRP (N=78,085) and haemoglobin (N=92,304).

The percentage of patients with abnormal biomarkers, values outside of the reference range, are presented in the table. The used reference ranges were: Creatinine (49-90 μmol/L for women, 60-110 μmol/L for men), Urea (2.5-6.4mmol/L for women, 2.5-7.5mmol/L for men), Sodium (136-145mmol/L), CRP (<10mg/L), Leukocytes (4.0-10.0x10^9/L), Haemoglobin (7.5-10.0mmol/L for women, 8.5-11.0mmol/L for men), Lactate (<2.0mmol/L).
